# Challenging the recessive paradigm of Mahvash disease: heterozygous phenotypes from a novel splice-site variant

**DOI:** 10.20945/2359-4292-2026-0005

**Published:** 2026-01-29

**Authors:** Francisco Martínez Bugallo, Gema García de la Rosa, Carol Prieto-Morín

**Affiliations:** 1 Human Genetic Unit, Department of Clinical Analysis Laboratory, University Hospital Nuestra Señora de Candelaria, Santa Cruz de Tenerife, Spain

**Keywords:** Glucagon receptor, pancreatic neoplasms, genetic predisposition to disease, splice site mutation, neuroendocrine tumors

## Abstract

Mahvash disease is a rare autosomal recessive condition caused by biallelic inactivating variants in the *GCGR* gene, impairing glucagon signaling and leading to alpha-cell hyperplasia and pancreatic neuroendocrine tumors (PNETs). Fewer than 20 cases have been reported, and the clinical impact of heterozygous variants remains unclear. Case Presentation: We report a family with a novel *GCGR* splice-site variant (c.1176+1_1176+7delGTGCCCG). The index case, a 61-year-old woman, presented with extensive pancreatic cystic disease and was found to be homozygous for the variant. She developed well-differentiated PNETs and underwent total pancreatectomy. Her sister, also homozygous, had similar clinical features and surgical history. In contrast, the heterozygous brother and two sons showed mild biochemical changes, such as elevated glucagon levels and small pancreatic cysts, without overt disease. The two homozygous sisters required pancreatic surgery followed by insulin and enzyme replacement therapy, whereas heterozygous carriers are currently being managed with biochemical and imaging surveillance. This family’s phenotype suggests a broader spectrum of *GCGR*-related disease. While homozygous individuals displayed classic Mahvash disease, heterozygotes exhibited subtle biochemical and structural pancreatic changes, indicating possible semidominant expression. These findings are consistent with emerging evidence that monoallelic receptor pathway mutations may produce mild or subclinical phenotypes. This case challenges the classical recessive model of Mahvash disease and highlights the potential for disease expression in heterozygous carriers. These findings suggest that heterozygosity is not entirely silent and underline the need to reconsider surveillance recommendations for *GCGR* heterozygotes.

## INTRODUCTION

Mahvash disease is a rare autosomal recessive disorder caused by inactivating mutations in the glucagon receptor gene (*GCGR*), leading to impaired glucagon signaling and compensatory pancreatic alpha-cell hyperplasia. Although fewer than 20 clinically confirmed cases have been reported worldwide ^(1-3)^, large-scale genomic studies suggest that the prevalence may be as high as four cases per million persons ^(2)^. This indicates that the condition is likely underrecognized rather than truly absent in the general population. Cases have been described in Asia, Europe, and North America, without evidence of geographical clustering. However, several families arose from consanguineous backgrounds ^(4,5)^, and accumulation of *GCGR* pathogenic variants in certain regions has been hypothesized to reflect founder effects or parental consanguinity ^(6)^.

Affected individuals typically show elevated plasma glucagon levels, pancreatic neuroendocrine tumors (PNETs) not associated with glucagonoma syndrome, and may develop diabetes, diarrhea, or pancreatitis ^(1-3)^. The clinical impact of heterozygous *GCGR* variants is unclear due to limited familial and experimental data. In murine models, *Gcgr*+/— animals display normal pancreatic morphology and do not develop α-cell hyperplasia or neuroendocrine tumors, in contrast to the fully penetrant phenotype observed in *Gcgr*-/— mice ^(1)^. However, in other recessive endocrine disorders, such as *MC4R* or *CYP21A2* deficiency, heterozygous carriers have been shown to manifest subtle clinical or biochemical phenotypes, suggesting that heterozygous states may not always be clinically silent.

Here, we describe a novel splice-site variant (c.1176+1_1176+7delGTGCCCG) segregated with clear phenotypic differences by zygosity, underscoring a discrepancy between animal models and human observations. These findings raise the possibility that heterozygous *GCGR* loss-of-function may exert clinical effects in humans, with implications for disease penetrance, surveillance, and counseling.

## CASE PRESENTATION

The index case, a 61-year-old woman, was initially evaluated due to the incidental discovery of extensive pancreatic cystic disease (over 50 cysts throughout the gland) along with radiological features suggestive of chronic pancreatitis. Despite these findings, she had no clinical signs of endocrine or exocrine pancreatic insufficiency. The imaging abnormalities and a family history of pancreatic neoplasia in her sister prompted further investigation. Initial genetic testing for CFTR and VHL was negative.

Genetic testing revealed homozygosity for a novel *GCGR* splice-site variant, NM_000160.5:c.1176+1_1176+7delGTGCCCG, identified via clinical exome sequencing. This 7-base deletion at the intron 12 donor site is predicted to disrupt splicing, leading to loss of receptor function. Absent from population databases and unpublished, its classification as likely pathogenic is supported by in silico predictions, familial segregation, and similarity to other *GCGR* loss-of-function variants ^(7,8)^.

The patient developed well-differentiated PNETs and underwent total pancreatectomy, splenectomy, and cholecystectomy. Histology confirmed well-differentiated neuroendocrine tumors. She required insulin and enzyme therapy, with suboptimal metabolic control. Serial biochemical follow-up revealed persistently elevated glucagon levels (208—219 pg/mL; normal range 50—150 pg/mL) and intermittently elevated chromogranin A (up to 196 ng/mL; normal range 0—100 ng/mL), consistent with the expected biochemical signature of Mahvash disease.

The 66–year–old sister, also homozygous for the *GCGR* variant, underwent duodenopancreatectomy, splenectomy, and cholecystectomy 14 years ago due to pancreatic tumors. She previously showed elevated chromogranin A levels and now remains on insulin and enzyme therapy with stable metabolic status. Both sisters exhibit the classic Mahvash disease phenotype, including PNETs and endocrine/exocrine dysfunction.

**Figure 1:**
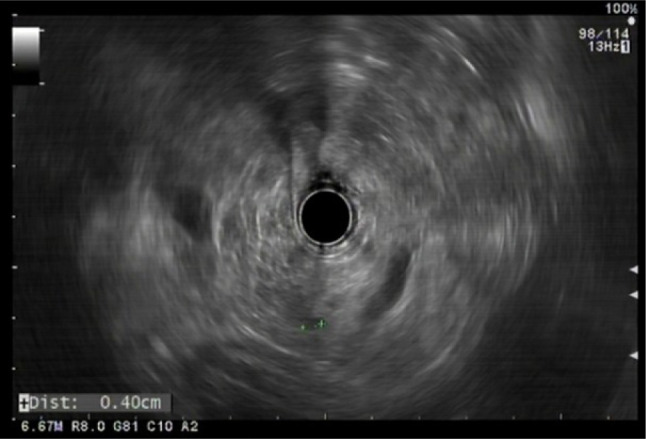
Endoscopic ultrasound of the heterozygous brother showing a 4 mm unilocular cyst in the posterior pancreatic body, with thin walls, no solid components, and no apparent communication with the main pancreatic duct.

The brother of both affected women, a 56–year–old man, was found to be heterozygous for the variant. He remains asymptomatic but has been under surveillance due to imaging findings of a subcentimetric intraductal papillary mucinous neoplasm (IPMN) without high–risk features. Imaging by endoscopic ultrasound (EUS) revealed a 4 mm cystic lesion in the pancreas, with no communication with the main pancreatic duct (Figure 1). His biochemical profile revealed a mild elevation in glucagon (178 pg/mL), though other tumor markers, including chromogranin A and pancreatic polypeptide, were within normal range. Surveillance has been carried out in accordance with the Kyoto guidelines for IPMN ^(9)^. Although these guidelines allow discontinuation of follow-up after 5 years in stable lesions <20 mm, extended surveillance with annual MRI was chosen in this case, given the presence of a familial *GCGR* variant and its potential clinical implications.

The index patient’s two sons, aged 36 and 31, are also heterozygous carriers. Both are clinically asymptomatic. The elder son had a normal MRI and normal biochemical parameters (glucagon 108 pg/mL; chromogranin A 60.31 ng/mL). In contrast, the younger son demonstrated a mild elevation in fasting glucagon levels (177 pg/mL), with normal chromogranin A and pancreatic polypeptide levels. Although no pancreatic lesions have been identified to date, this subtle increase in glucagon raises the possibility of early or subclinical alpha–cell proliferation in heterozygous carriers. He is currently undergoing conservative annual MRI, biochemical surveillance, and additional renal ultrasound to assess for possible cystic disease as observed in other relatives; however, the long–term monitoring strategy for heterozygous carriers has yet to be defined and may require adjustment as further data emerge.

The 86–year–old mother of the three siblings is heterozygous for the *GCGR* variant and, despite being asymptomatic, shows chronically elevated pancreatic enzymes without imaging evidence of lesions. No hormonal tests have been performed. Differential diagnoses such as chronic pancreatitis, subclinical ductal obstruction, medication effects, or age–related pancreatic changes were considered, although none were clinically suspected. Given her age and stable clinical course, further extensive investigations were not pursued. The deceased father was not genetically tested.

## DISCUSSION

This family provides evidence that *GCGR*–related pathology may extend beyond the classical autosomal recessive model. While homozygous individuals clearly show the full Mahvash disease phenotype, the presence of pancreatic cysts and mildly elevated glucagon in heterozygotes suggests a possible semidominance effect. These findings align with emerging observations that some recessive conditions may manifest subtly in heterozygous carriers, particularly those involving receptor or hormonal pathways ^(10)^.

Consistent with previous reports describing *GCGR* variants in consanguineous families ^(6)^, the segregation pattern observed in this family — two homozygous daughters, a heterozygous mother, and an untested deceased father — strongly suggests paternal carrier status. Given the family’s origin from a small, isolated village of around 400 inhabitants, distant consanguinity cannot be excluded.

The biochemical findings in this family support a spectrum of alpha–cell activity, even without evident neuroendocrine tumors. The index case and her homozygous sister showed significant elevations in glucagon and chromogranin A, consistent with Mahvash disease. Heterozygous relatives had mild glucagon increases, possibly indicating early or subclinical alpha–cell hyperplasia. Normal chromogranin A and pancreatic polypeptide levels in heterozygotes suggest no active tumor development. This pattern indicates that glucagon elevation may precede structural changes and could serve as an early marker for monitoring at–risk heterozygous individuals. Notably, while heterozygous *Gcgr*+/— mice show no pancreatic abnormalities, our findings in human carriers suggest that subtle biochemical or structural changes may still occur, highlighting an important divergence between animal models and human disease expression. Whether more subtle functional changes occur in heterozygous animals has not been systematically evaluated, and thus cannot be excluded.

Recent work by Kuiper and cols. ^(11)^ also described a family with a heterozygous *GCGR* variant (p.Ser152Phe) in which one carrier developed PNETs. Interestingly, somatic *MEN1* mutations were identified in the tumors of that individual, suggesting that reduced glucagon receptor signaling may create a permissive state that, when combined with additional genetic events, promotes neoplastic transformation. This observation supports the notion that heterozygous *GCGR* loss–of–function alone may not invariably lead to clinically significant disease, but can predispose to pathological changes under specific conditions. A separate report by Deng and cols. ^(12)^ described a patient labelled as “heterozygous” who carried two *GCGR* variants (c.1345C>T and c.922C>T). One of these is a truncating variant likely to be pathogenic, while the other is a missense variant currently classified as a variant of uncertain significance, with limited evidence supporting a pathogenic role. In addition, segregation analysis was not performed, leaving it unclear whether the two variants are located on the same allele or on different alleles. Clinically, the patient showed a diffusely enlarged pancreas with heterogeneous enhancement on imaging and developed pancreatic neuroendocrine tumors. However, because the diagnosis relied primarily on clinical and imaging findings rather than on definitive genetic evidence, the underlying genetic status and mode of inheritance in this case remain highly uncertain, and it provides only limited insight into the potential consequences of *GCGR* heterozygosity.

These findings may suggest that clinical surveillance strategies for individuals heterozygous for *GCGR* pathogenic variants may need reconsideration. Although current guidelines assume complete recessivity and don’t recommend follow–up for carriers, our observations in this family indicate that a cautious conservative monitoring approach could be considered. In particular, we propose—based on this limited experience—annual clinical and biochemical evaluation, with special attention to fasting glucagon levels, complemented by periodic pancreatic imaging using MRI or EUS. The optimal frequency and duration of imaging are not yet established, but an initial interval of 1–2 years appears reasonable and safe as a provisional approach until further evidence becomes available. This schedule could then be individualized over time according to the evolution of pancreatic findings and the patient’s overall clinical course. From the radiological perspective, recent correspondence has emphasized the importance of carefully interpreting subtle cystic or ductal changes in carriers, as these may represent early or subclinical manifestations of disease ^(13)^. We emphasize that these recommendations are hypothetical and derived from a single family, and are intended mainly to stimulate further discussion and research on the management of heterozygous *GCGR* carriers.

Although the long–term risks for heterozygous carriers are not yet defined, our findings may suggest a potential for progression from isolated biochemical changes to structural pancreatic abnormalities. The development of clinically significant complications, such as α–cell hyperplasia or neuroendocrine tumors, appears uncommon but cannot be excluded, particularly if additional modifying factors are present. Thus, carriers should not be considered entirely unaffected, but their risk profile remains far lower and less predictable than that of homozygous individuals.

A recent case series by Gild and cols. ^(5)^ reviewed *GCGR* variants reported in Mahvash disease patients, including missense, nonsense, frameshift, and in–frame deletion mutations. These variants were observed in both homozygous and compound heterozygous states. These biallelic loss–of–function mutations often lead to the full disease phenotype. Our findings align with this and further suggest that even monoallelic loss of *GCGR* function may cause pancreatic or biochemical changes under certain conditions. Although the functional impact of this specific variant has not yet been confirmed, it is analogous to other loss–of–function mutations in *GCGR* that lead to absent receptor expression or activity ^(1,4)^.

As with many reports on rare genetic disorders, this study is inherently limited by the small number of affected individuals available for analysis — here restricted to a single family. The scarcity of published data on heterozygous *GCGR* carriers reflects a broader gap in the field rather than a shortcoming of this report. Another limitation is that alternative explanations for the incidence of pancreatic cysts in this family, such as unrelated genetic predispositions or shared environmental factors, cannot be fully excluded. However, the clear segregation of both biochemical abnormalities and cystic findings with *GCGR* zygosity argues in favor of a causal role of the variant, making these alternatives less likely. Taken together, these observations provide meaningful insights into the potential clinical impact of heterozygous variants. Rather than offering definitive conclusions, our report underscores the need for further investigations and may serve a foundation for developing surveillance strategies in this previously overlooked group.

## CONCLUSION

In conclusion, this report describes a family with a novel *GCGR* splice–site variant segregated in both homozygous and heterozygous individuals, with phenotypes ranging from classic Mahvash disease to subtle biochemical or structural pancreatic changes. The consistent detection of mild hyperglucagonemia in heterozygotes, together with occasional cystic findings, suggests that *GCGR*–related pathology may extend beyond the strictly recessive model and include a broader phenotypic spectrum. Our observations raise the possibility that heterozygous carriers might manifest a mild phenotype compatible with a dominant pattern of low penetrance and variable expressivity, although additional studies are needed to confirm this hypothesis and better define its clinical implications, including surveillance strategies for carriers.

## Data Availability

datasets related to this article will be available upon request to the corresponding author.
